# Multicentric Reticulohistiocytosis Exhibiting Positive HLA-B*07 and HLA-B*08: A Case Report

**DOI:** 10.3390/medicina56090456

**Published:** 2020-09-08

**Authors:** Elena Rezuș, Maria Alexandra Burlui, Anca Cardoneanu, Danisia Haba, Mihai Danciu, Romică Sebastian Cozma, Ciprian Rezuș

**Affiliations:** 1 Department of Rheumatology and Physiotherapy, Faculty of Medicine, “Grigore T. Popa” University of Medicine and Pharmacy, 16 Universității Street, 700115 Iași, Romania; elena.rezus@umfiasi.ro (E.R.); anca.cardoneanu@umfiasi.ro (A.C.); 2 Department of Rheumatology, Clinical Rehabilitation Hospital, 14 PantelimonHalipa Street, 700661 Iași, Romania; 3 Department of General and Dental Radiology, Faculty of Dental Medicine, “Grigore T. Popa” University of Medicine and Pharmacy, 16 Universității Street, 700115 Iași, Romania; danisia.haba@umfiasi.ro; 4 Department of Morphopathology, Faculty of Medicine, “Grigore T. Popa” University of Medicine and Pharmacy, 16 Universității Street, 700115 Iași, Romania; mihai.danciu@umfiasi.ro; 5 Department of Otorhinolaryngology, Faculty of Medicine, “Grigore T. Popa” University of Medicine and Pharmacy, 16 Universității Street, 700115 Iași, Romania; sebastian.cozma@umfiasi.ro; 6 Department of Internal Medicine, Faculty of Medicine, “Grigore T. Popa” University of Medicine and Pharmacy, 16 Universității Street, 700115 Iași, Romania

**Keywords:** multicentric reticulohistiocytosis, human leucocyte antigen, major histocompatibility complex, sacroiliac joints, inflammatory arthritis, autoantibody

## Abstract

Multicentric reticulohistiocytosis (MRH) is a rare cause of destructive inflammatory arthritis involving both small, as well as larger joints. We report the case of a 40-year-old Caucasian female with a family history of neoplasia who was referred to our service witha two-month history of inflammatory joint pain. On examination, the patient had inflammatory arthritis, mainly involving the peripheral joints, sacroiliac joint pain, and numerous papulonodular mucocutaneous lesions, including periungual “coral beads”. Imaging tests revealed erosive arthritis with synovitis and tenosynovitis, sacroiliac joint changes, as well as papulonodular mucosal lesions in the nasal vestibule, the oropharyngeal mucosa, and supraglottic larynx. She tested positive for HLA-B*07 (Human Leukocyte Antigen B*07) and HLA-B*08, ANA (antinuclear antibodies), RF (rheumatoid factor), anti-Ro52, anti-SSA/Ro, and anti-SSB/La antibodies. The skin biopsy was suggestive of MRH, showing a histiocyte infiltrate and frequent giant multinucleated cells. The patient exhibited favorable outcomes under Methotrexate, then Leflunomide. However, she displayed worsening clinical symptoms while under Azathioprine. To our knowledge, this is the first case of MRH to exhibit positive HLA-B*07 together with HLA-B*08. The rarity of MRH, its unknown etiology and polymorphic clinical presentation, as well as its potential neoplastic/paraneoplastic, and autoimmune nature demand extensive investigation.

## 1. Introduction

Multicentric reticulohistiocytosis (MRH) is a multisystem granulomatous non-Langerhans cell histiocytosis (non-LCH) and a very rare cause of destructive (erosive) inflammatory arthritis [1]. MRH has been classified as a C-Group histiocytosis (non-LCH involving both skin and mucosae), in the subgroup with notable systemic involvement [2]. While the joint involvement often presents with symmetric and destructive arthritis, cutaneous eruptions include pathognomonic periungual “coral beads” and a “vermicular” aspect of the lesions bordering the nostrils [3].

The infiltrate of CD163+ (cluster of differentiation 163), CD68R+, CD1a− large mono- to multinucleated cells with ground-glass cytoplasmin the biopsy specimens of the affected tissues depicts the histological mark of MRH [1,3].

It has been hypothesized that patients with MRH may harbor mutations of the oncogene BRAF (B-Raf Proto-Oncogene Serine-Threonine Kinase), as well as MAP2K1 (Mitogen-Activated Protein Kinase Kinase 1), which are both involved in the RAS-MAPK (Ras/Mitogen-Activated Protein Kinase) signaling pathway and could be relevant as therapeutic targets [4,5]. Murakami et al. examined biopsy specimens from 15 subjects with histiocytoses (two patients with MRH and 13 patients with LCH) from a genetic perspective and identified MAP2K1 mutations (similar to LCH) together with an FGFR1 (fibroblast growth factor receptor 1) tyrosine kinase fusion (which were not detected in LCH) [4]. The authors proposed that MRH be classified as a neoplastic condition, given that its pathomechanism may involve the activation of the RAS-MAPK pathway [6]. Indeed, associations between MRH and various types of neoplasia and autoimmune diseases have been reported [5,7]. Nevertheless, the neoplastic/paraneoplastic andautoimmune nature of the disease remains a matter of debate [1,2,3,4,5,6,7].

The diagnosis of MRH requires a multidisciplinary clinical, pathological, and radiological approach [1,2,3,4,5,6,7]. Herein, we present a case of MRH exhibiting both typical clinical changes, as well as certain distinct traits. We have obtained approval from the Ethics Committee for the present work (10371/29.04.2020).

## 2. Case Report

We report the case of a 40-year-old Caucasian female who was referred to our clinic for a two-month history of inflammatory joint pain, xerostomia, xerophtalmia, and skin changes. Her family history was significant for neoplasia—sister with synchronous tumors of the digestive tract (colon and stomach) and grandfather with nasopharyngeal carcinoma.

**Figure 1:**
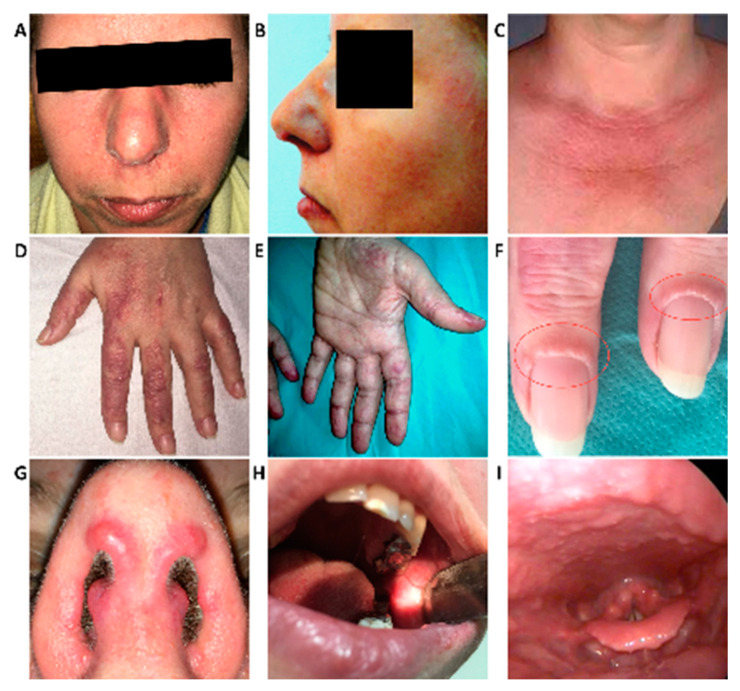
Mucocutaneous changes. Papulonodular lesions and diffuse erythema—forehead, nose, cheeks, upper lip (**A**,**B**); dermatomyositis-like rash on the chest (**C**); Papulonodular lesions—dorsum and palmar surface of hands/fingers (**D**,**E**); Periungual “coral beads” (**F**); “Vermicular” erythematous lesions bordering the nostrils, papulonodular lesions in the nasal vestibule (**G**); Papulonodular lesions at the level of the oral mucosa (**H**); Endoscopic aspect of the larynx—papulonodular lesions (**I**).

On examination, she had inflammatory arthritis mainly involving peripheral joints (small joints of the hands, wrists, and knees), pain in the lower back and both sacroiliac joints, numerous papulonodular mucocutaneous lesions, including periungual “coral beads”, a dermatomyositis-like rash on the upper thorax (Figure 1), as well as yellowish papular plaques on the lower eyelids (suggestive of *xanthelasma palpebrarum*). The manual muscle testing was normal (5/5), both proximally and distally.

The patient also displayed high erythrocyte sedimentation rate (ESR) and C-reactive protein (CRP) levels. The immunological findings included low-positive rheumatoid factor (RF) together with high titers of antinuclear antibodies (ANA), anti-Ro52, anti-SSA/Ro, and anti-SSB/La, as well as positive HLA-B*07 and HLA-B*08 (Human Leucocyte Antigen; other HLA-B alleles, including B*27 were negative). She was tested for a variety of other autoantibodies such as anti-CCP (cyclic citrullinateid peptide), anti-dsDNA (double stranded DNA), anti-Sm (Smith), anti-nucleosome, anti-SRP (signal recognition particle), anti-Mi2, anti-synthetase, anti-U1RNP (ribonucleoprotein), anti-RNP/Sm, anti-topoisomerase I, and anti-centromere, which were all negative.

The endoscopic examination of the upper airways identified papulonodular lesions in the nasal vestibule, pharyngeal mucosa, supraglottic larynx, and arytenoid mucosa (Figure 1, Video S1: Endoscopy).

**Figure 2:**
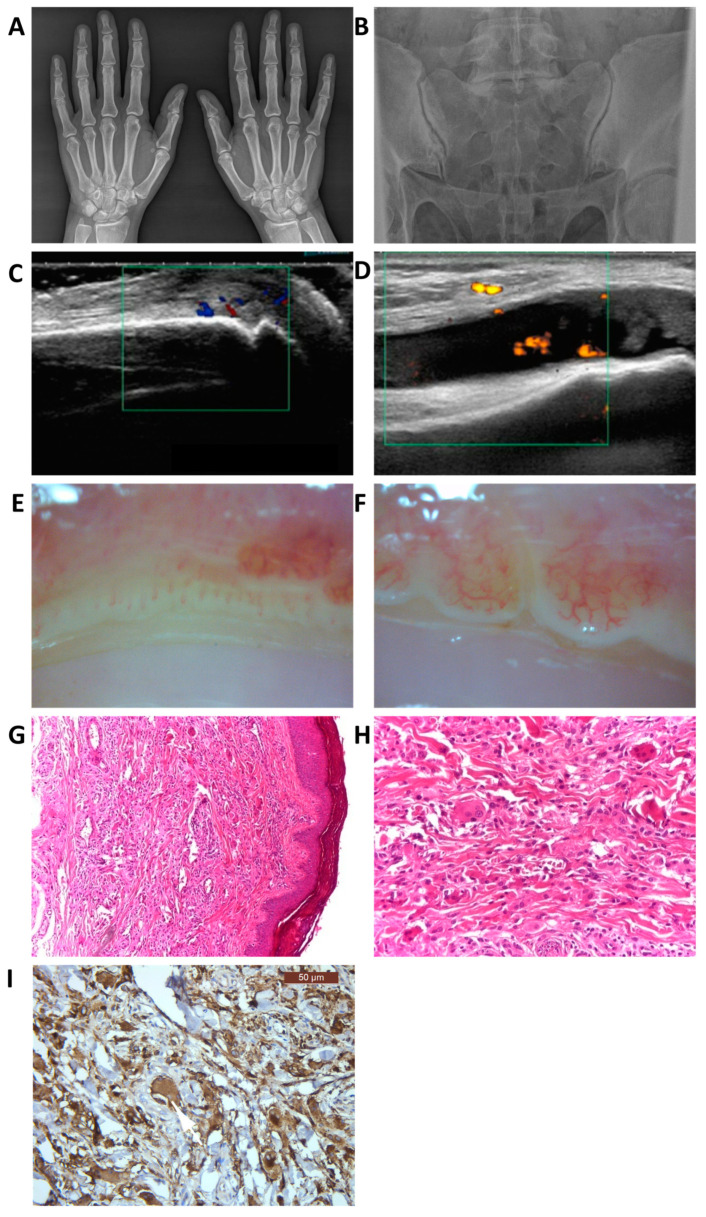
Paraclinical findings. Hand X-rays revealing an erosion at the level of the right DIP3 (distal interphalangeal joint) and subchondral cysts in the thumb MCP (metacarpophalangeal) joints on both hands (**A**); Sacroiliac joint X-rays showing bilateral joint space narrowing, subchondral osteocondensation, and irregular joint contour on both the iliac and the sacral sides (more obvious in the right sacroiliac joint) (**B**); Joint ultrasound: proliferative synovitis with positive Doppler signal—3rd MCP joint, right hand (**C**), proliferative synovitis—left knee (**D**); Capillaroscopic examination of the periungual area (200X magnification): areas of normal capillary density and morphology intercalated with papulonodular lesions (**E**,**F**); Skin biopsy: histological aspect in hematoxylin-eosin staining X4 (**G**) and X10 (**H**): mononuclear cell infiltrate and isolated lymphocytes with frequent giant multinucleated cells located between the dermal collagen fibers; Immunohistochemistry: the mono- and multinucleated cells were positive for CD68 (**I**).

We performed hand X-rays, which revealed an erosion at the level of the right DIP3 (distal interphalangeal joint) and subchondral cysts in the thumb MCP (metacarpophalangeal) joints on both hands (Figure 2). The joint ultrasound revealed subcutaneous inflammatory nodules, synovitis in the small joints of the hands as well as both knees (up to grade 4 in the MCP joints), and tenosynovitis (up to grade 4 in both extensor and flexor compartments of the hands) with active vascularization, and erosions in the small joints of the hands.

The sacroiliac X-rays showed bilateral joint space narrowing, subchondral osteocondensation, and irregular joint contour on both the iliac and the sacral sides (more obvious in the right sacroiliac joint) (Figure 2). We did not find notable changes in the spine.

The capillaroscopic examination described papulonodular lesions with a vascular center intercalated with areas of normal capillary density and morphology (Figure 2).

The skin biopsy was suggestive of MRH, revealing a mononuclear cell infiltrate and isolated lymphocytes with frequent giant multinucleated cells located between the dermal collagen fibers. On immunohistochemistry, the mono- and multinucleated giant cells were positive for CD68 and negative for S100 (Figure 2).

Following diagnosis, the patient was started on Methotrexate (up to 20 mg/week) and Methylprednisolone 16 mg/day, which significantly ameliorated joint involvement (no inflammatory pain or synovitis on ultrasound), mucocutaneous changes (disappearance of mucosal lesions, disappearance or amelioration of the cutaneous eruptions), and systemic inflammation (normal ESR and CRP).

She was able to gradually withdraw from glucocorticoids after six months of use and remained stable under Methotrexate 20 mg/week until one year later when she suffered a relapse of both inflammatory arthritis and mucocutaneous lesions. Following this episode, Methotrexate was stopped and the patient was started on Azathioprine 100 mg/day and Methylprednisolone 32 mg/day under which she developed worsening joint involvement (reappearance of inflammatory arthritis, with active synovitis and tenosynovitis and new erosions in the right wrist), as well as new mucocutaneous lesions. Nonetheless, ESR and CRP levels remained within normal limits. Consequently, the patient was started on Leflunomide 20 mg/day, which led to an improvement of the clinical symptoms. She was able to stop Methylprednisolone in November 2019, and has remained stable and corticoid-free ever since.

The patient was also referred to an ophthalmologist for xeropthalmia. Other than abnormal values of the Schirmer’s test (3.5 mm), no other ocular anomalies were noted.

**Figure 3:**
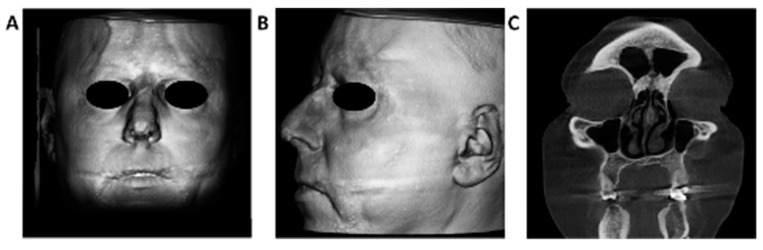
Cone-beam CT. 3D facial reconstruction showing papulonodular lesions on the forehead, nose, cheeks, upper lip, chin (**A**,**B**); Inflammatory changes at the level of the ethmoidal mucosa on the right side (**C**).

In order to exclude malignancy as a possible trigger for the appearance of MRH, we performed several imaging tests. The chest Xrays, thoracoabdominal computed tomography (CT), and ultrasound of the abdomen, pelvis, neck, parotid, thyroid, and parathyroid glands were unremarkable. The cone-beam CT showed inflammatory changes at the level of the ethmoidal mucosa on the right side, without any other pathological findings (Figure 3).

Two years after diagnosis, we performed a pelvic CT scan, which identified a uterine nodule together with ovarian cystic lesions. These findings prompted a detailed assessment by a gynecologist, including a biopsy, further imaging tests, and breast examination, which excluded underlying neoplasia. Following investigations, the lesion was classified as benign (uterine fibroid).

## 3. Discussion

With around 300 cases reported to date, MRH is a very rare disease that remains poorly understood [1]. Our patient exhibited typical skin and joint involvement as well as a characteristic histological aspect of the skin biopsy. However, she also demonstrated sacroiliac joint changes.

Sacroiliac joint involvement has previously been reported in MRH [3,5], yet it is not regarded as a cardinal sign of the disease [4]. Noteworthy, the majority of case reports in the currently available literature do not mention investigating the sacroiliac joints. Whereas HLA-B*B27 is an immunogenetic hallmark of spondyloarthritides (with a predilection for ankylosing spondylitis), several other HLA alleles could also be associated with these diseases. In this regard, HLA-B*07 has been listed among the alleles, which potentially raise the susceptibility for developing spondyloarthropathies [8]. Our patient tested positive for HLA-B*07, albeit not meeting the diagnostic criteria for ankylosing spondylitis or other spondyloarthritides.

In their MRH patient, Mead et al. found erosions with a “punched-out” aspect at the level of the PIP and DIP joints together with osteosclerosis, involving the PIP, DIP, and both thumb interphalangeal joints [9]. Similarly, our patient exhibited erosive lesions (DIP3—right hand). Moreover, she presented with symmetrical joint involvement (subchondral cysts in the thumb MCP joints on both hands).To our knowledge, this is the first case of MRH to exhibit HLA-B*07 together with HLA-B*08 positivity. El-Gabalawy et al. examined the expression of HLA alleles in a cohort of patients with recent-onset inflammatory arthritis (active synovitis) compared to controls [10]. The authors’ findings included 27% HLA-B*08 positivity in a subgroup with undifferentiated arthritis (UA—patients who did not meet the criteria for rheumatoid arthritis or spondyloarthritides). Whereas it was mentioned that one patient in the UA group had been diagnosed with MRH, El-Gabalawy et al. did not provide details pertaining to the HLA characteristics found in that specific case [10].

The immune disturbance we found in our patient included positive ANA, anti-Ro52, anti-SSA/Ro, anti-SSB/La antibodies, and low-positive RF. Some authors found no inflammatory syndrome or autoantibodies in their patients [11,12,13], while others reported positive ANA and anti-CCP antibodies [6,14]. However, the issue of MRH possibly overlapping with autoimmune rheumatic conditions remains a matter of debate [11]. Positive anti-Ro52 antibodies have been found in immune-inflammatory rheumatic conditions, such as idiopathic inflammatory myopathies, systemic lupus erythematosus, systemic sclerosis, and Sjögren’s syndrome [15,16], as well as certain malignancies [17,18]. In regard to our patients, we have not found evidence of underlying neoplasia to date. Nevertheless, she met the classification criteria for Sjögren’s syndrome (positive anti-SSA/Ro antibodies and Schirmer’s test).

The risk factors for Sjögren’s syndrome include the involvement of certain MHC (major histocompatibility complex) class II genes [19]. In this respect, HLA-DQ and DR have been shown to be especially relevant, with DR2 and DR3 associating with specific autoantibody production in Caucasian subjects [20]. It has been stated that MICA*008 (MHC class I chain-related gene A), an HLA-independent factor, may be related to the development of primary Sjögren’s syndrome [21]. Moreover, MICA*008 is in linkage disequilibrium with HLA-B*08:01 [21]. However, it has been shown that MICA*008 could be connected to primary Sjögren’s syndrome independently from the loci that are in linkage disequilibrium with this specific factor [21].

Currently, there is no universally accepted treatment protocol for multicentric reticulohystiocytosis; some patients responding to synthetic disease-modifying antirheumatic drugs such as Methotrexate, Leflunomide, Azathioprine, Cyclosporine A [3], while others demonstrating positive outcomes under anti-TNFα (Tumor Necrosis Factor α) therapy, such as Adalimumab and Infliximab [22], bisphosphonates, or chemotherapy [3,23]. Our patient exhibited a good response to Methotrexate (initially—both skin as well as joint involvement), then Leflunomide. Nonetheless, treatment with Azathioprine did not lead to positive outcomes in her case. Disease activity and severity, as well as the factors potentially influencing multicentric reticulohystiocytis patients’ response to the treatment are yet to be established.

## 4. Conclusions

Mainly due to the rarity of the disease, MRH continues to pose diagnostic and therapeutic challenges for clinicians. The low number of cases reported in the currently available literature leaves many unknowns regarding the pathogenesis and the optimal treatment of this rare (yet potentially destructive) cause of inflammatory arthritis.

It is yet to be determined whether or not certain immunogenetic factors (including HLA-B alleles) are common and relevant in MRH. Moreover, the prevalence, severity, and possible diagnostic or prognostic value of sacroiliac joint involvement in these patients remaina matter of further investigation.

## Supplementary Materials

The following are available online at https://www.mdpi.com/1010-660X/56/9/456/s1, Video S1: Endoscopy.
